# COVID-19: Role of Nutrition and Supplementation

**DOI:** 10.3390/nu13030976

**Published:** 2021-03-17

**Authors:** Fiorenzo Moscatelli, Francesco Sessa, Anna Valenzano, Rita Polito, Vincenzo Monda, Giuseppe Cibelli, Ines Villano, Daniela Pisanelli, Michela Perrella, Aurora Daniele, Marcellino Monda, Giovanni Messina, Antonietta Messina

**Affiliations:** 1 Department of Clinical and Experimental Medicine, University of Foggia, 71122 Foggia, Italy; fiorenzo400@gmail.com (F.M.); francesco.sessa@unifg.it (F.S.); anna.valenzano@unifg.it (A.V.); rita.polito@unicampania.it (R.P.) giuseppe.cibelli@unifg.it (G.C.); daniela.pisanelli82@gmail.com (D.P.); perrellamichela@tiscali.it (M.P.); 2 Department of Medical Sciences and Advanced Surgery, Università degli Studi della Campania “Luigi Vanvitelli”, 80138 Naples, Italy; 3 Department of Experimental Medicine, Section of Human Physiology and Unit of Dietetics and Sports Medicine, Università degli Studi della Campania “Luigi Vanvitelli”, 80131 Naples, Italy; vincenzo.monda@unicampania.it (V.M.); ines.villano@unicampania.it (I.V.); antonietta.messina@unicampania.it (A.M.); 4 CEINGE Biotecnologie Avanzate S.C. a r.l., 80131 Napoli, Italy; aurora.daniele@unicampania.it

**Keywords:** nutrition, COVID-19, dietary supplement, COVID-19 and diet

## Abstract

At the end of 2019, a new coronavirus (COVID-19) appeared on the world scene, which mainly affects the respiratory system, causing pneumonia and multi-organ failure, and, although it starts with common symptoms such as shortness of breath and fever, in about 2–3% of cases it leads to death. Unfortunately, to date, no specific treatments have been found for the cure of this virus and, therefore, it is advisable to implement all possible strategies in order to prevent infection. In this context, it is important to better define the role of all behaviors, in particular nutrition, in order to establish whether these can both prevent infection and improve the outcome of the disease in patients with COVID-19. In the literature, it is widely shown that states of malnutrition, overweight, and obesity negatively affect the immune system, leading to viral infections, and several studies have shown that nutritional interventions can act as immunostimulators, helping to prevent viral infections. Even if several measures, such as the assumption of a specific diet regimen, the use of dietary supplements, and other similar interventions, are promising for the prevention, management, and recovery of COVID-19 patients, it is important to highlight that strong data from randomized clinical trials are needed to support any such assumption. Considering this particular scenario, we present a literature review addressing several important aspects related to diet and SARS-CoV-2 infection, in order to highlight the importance of diet and supplementation in prevention and management of, as well as recovery from COVID-19.

## 1. Introduction

Since its appearance at the end of 2019, coronavirus disease 2019 (COVID-19) has immediately shown a high rate of transmission, forcing the World Health Organization (WHO) to declare in March 2020 that this unknown coronavirus, named severe acute respiratory syndrome coronavirus 2 (SARS CoV-2), can be characterized as a pandemic [1,2]. In the past twenty years, coronavirus (CoV) infections have raised many concerns for public health. In fact, in 2002, there was the first epidemic due to a coronavirus, originating in China, which was related to a severe acute respiratory syndrome, called SARS-CoV [3]. Subsequently, in 2012, a new viral outbreak with characteristics similar to SARS-CoV was observed in the Middle East (Qatar, Jordan, Saudi Arabia, and the United Arab Emirates), in Europe (UK, France, and Italy), and in Africa, and was called Middle East respiratory syndrome (MERS) [4]. 

COVID-19 patients can develop the disease in different forms: They can be asymptomatic, have mild symptoms, or they can also have severe symptoms that can lead to hospitalization, and in severe cases death [5]. The most serious clinical conditions are characterized by acute respiratory distress syndrome (ARDS), cardiac insufficiency, and septic shock [6,7,8], and this causes tissue damage at the alveolar level, generating pathological tissue alterations, hyperplasia, and infiltration. In addition, the existence of different comorbidities in subjects infected by SARS-CoV-2 may increase the response of the immune system, exacerbating the risk of adverse effects and mortality [9,10,11]. Indeed, the systemic inflammation found in individuals suffering from non-communicable diseases (such as diabetes and arterial hypertension) is strictly related to severe symptoms after the SARS-CoV-2 infection [12,13]. 

Nutritional influence on the immune system has been well documented in the literature [14]. In general, where practicable, an efficient way to reduce the risk of viral infections is to regulate the actions of the inflammation mediators through adaptable risk factors such as diet, exercise, and healthful lifestyle habits. [15]. Adopting a consistent, long-term dietary pattern is the only way to benefit human health. On the contrary, the adoption of an unhealthy lifestyle (i.e., worsening the diet regimen and reducing physical activities) is associated with a high level of oxidative stress, which could lead to the development of non-communicable diseases (NCDs). [16]. Admittedly, it is well described that the diet regimen strongly influences the immune system responses [17]. Some reports [18] have indicated that a high body mass index (BMI) or excessive adiposity may be risk factors for complications during COVID-19 infection [19]. This may be caused by the presence of different pulmonary diseases in overweight and obese populations compared to healthy weight subjects. [20]. Moreover, obese patients may be considered at greater risk of developing severe COVID-19 disease for the presence of other comorbidities that could impair their heart or pulmonary function [21], just like non-obese subjects with those risk factors. For these reasons, particularly in this pandemic period, it is important to maintain a weight and body composition in line with the international recommendations for stature and sex [22].

In addition, recent studies have reported the positive influence of nutritional status and food intake in COVID-19 patients. However, in consideration of the novelty of the SARS-CoV-2 infection, studies on the effects of certain nutrients are scarce, and in some investigations these data are obtained from ecological studies [23]. The aim of this review was to provide an overview regarding the possible effects of nutrition and supplementation not only in the prevention of COVID-19 and taking care of these patients, but in the management of COVID-19 survivors.

## 2. Methods

### 2.1. Eligibility Criteria

All case reports, letters to editors, case series, reviews, retrospective and prospective studies, which focused on SARS-CoV-2 and “nutrition” OR “diet” OR” “supplement”, were included. The search was limited to human studies.

### 2.2. Search Criteria

A systematic literature review and critical evaluation of the collected studies were carried out. An electronic search of PubMed from the inception of this database to the 31 January 2021 was performed. Search words were: (“coronavirus” OR “SARS-CoV-2” OR “COVID-19” OR “severe acute respiratory syndrome coronavirus 2”) AND (“nutrition” OR “diet” OR “supplement”). 

## 3. Results

In this review, using the keywords reported in the methods, we selected 36 publications indexed on PubMed regarding the topics COVID-19 and nutrition. After an initial analysis we took into consideration 16 articles (Table 1).

### 3.1. COVID-19 and Nutrition

Numerous scientific studies show that adequate nutrition is required for all cells, including those of the immune system, to function at their best [17]. An “activated” immune system additionally enhances energy demands during the SARS-CoV-2 infection, with an increased basal metabolic rate. Therefore, optimized nutrition for the best immune outcomes would be one that supports immune cell function by allowing them to engage robust responses to pathogens, but also to improve the responsiveness when appropriate, avoiding any underlying chronic inflammation.

As reported by Clader et al., the current status of the SARS-CoV-2 infection and COVID-19 adverse effects, combined with the data related to morbidity and mortality figures of other respiratory infections, in general underline the concept that improving nutrition status should not be considered sufficient particularly when it is adopted without other treatments. Moreover, considering that new pathogens responsible for influenza continually emerge, and other outbreaks of new viruses could occur, it is necessary to develop new effective interventions in order to reduce their adverse effects. In this way, additional safe and convenient strategies are requested to improve the immune system response. One convincing strategy is to provide an adequate dietary regimen in order to stimulate the immune system. [3].

In another important review, the authors concluded that a poor nutritional status appears to be a predictor of mortality in acute viral infection and critical disease, particularly for elderly subjects. The recommendations for dietary supplementation in critical illness are applied to COVID-19 patients who require intensive care unit (ICU) support. In addition, nutritional and dysphagia monitoring is recommended for recovering subjects, and thus, long-term infection outcomes have not yet been explored [24].

Cena et al. [25] suggest that to improve the efficiency of the immune system, it would be advisable to include specific foods in the diet as good sources of antioxidants, such as fresh fruit and vegetables, soy, nuts [38], and omega-3 fatty acids all being low in saturated fats and trans fats [39]. In addition, moderate diet regimen is suggested for obese/overweight subjects and diabetic patients [40]. Dietary status seems to be an important factor that influences the outcome of COVID-19 patients, but to date no clear information is available on the effectiveness of early nutritional integration in COVID-19 patients [26,27,28,35,36]. Zhang and Liu recently proposed a comprehensive list of nutritional supplements with possible beneficial effects in COVID-19 patients based on clinical studies and in vitro studies [41]. Nevertheless, to date, no paper has been published focusing on the thematic of how to improve the current recommendations and guidelines for nutrition to this poorly known disease. Aman et al. in their brief report suggested the use of nutritional status to measure resilience towards destabilization during the COVID-19 pandemic [29].

Optimized dietary assumption influences the immune system through the modification of signaling molecules, influencing the cellular activation, and gene expression. In this regard, various nutrients are also determinants of gut microbial composition and shaping immune responsiveness in the body. Hence, several studies suggest that reinforcing the immune system represents one sustainable way to improve the possibility to survive in this pandemic situation.

In line with the hypothesis by Aman et al., another author added that this period could be looked upon as an opportunity [29]. The onus is on those in authority to promote and facilitate a nutritional culture on the population to achieve behavior change and on healthcare professionals to embed nutritional care into routine practice. None of these measures are new or groundbreaking, but perhaps have not been at the forefront in recent years. If we are collectively able to implement and sustain these strategies during and after this pandemic, there may be at least one positive legacy of COVID-19. Necessity is, after all, the mother of (re)invention [30]. In another review the authors declare that it is important have a diet based primarily on fresh food such as fruit, vegetables, whole grains, low-fat dairy, and healthy fats (olive oil and fish oil), limiting the intake of sugary drinks, high-calorie, and high-salt foods [42].

### 3.2. COVID-19 and Dietary Supplements

Virus infections are marked by impairment of the immune system with a subsequent insufficient micronutrient reserve, as shown in a recent review, when several substances, such as vitamins (including vitamins A, B6, B12, C, D, E, and folate) and other elements (i.e., zinc, iron, selenium, magnesium, and copper), are deficient [43]. As widely demonstrated in the literature, and also discussed in several reviews, the intake of different substances such as essential fatty acids, linoleic acids, essential amino acids, and the vitamins and minerals mentioned above can improve the immune response, especially where immunity can also be conditioned by deficiencies as in the case of viral infections [44]. Three recent reviews have discussed how adequate nutritional intake, combined with the integration of different functional foods, helps maintain optimal levels in the human body by improving various aspects of the immune system [45,46,47]; in addition, in two other recent reviews, several observational studies and clinical studies have been taken into consideration, in which it has been shown that vitamin D supplementation reduces the risk of influenza, while others do not [48,49]. In contrast, in a retrospective cohort study of 201 patients with confirmed COVID-19 pneumonia admitted to Wuhan Jinyintan Hospital in China between 25 December 2019 and 26 January 2020, it has been well described that one of the main risk factors for COVID-19 is an insufficient immune response [50] and, therefore, it is appropriate to discuss the protective role of diet regimen and supplement assumption in the prevention of both seasonal infections and of SARS-CoV-2. Particularly, the scientific community is being called on to carry out new research in this challenging field.

Vitamin D insufficiency occurs in approximately 50% of the world’s population [51]. In a cross-sectional study it was shown that the high prevalence of vitamin D deficiency represents an important concern for public health because hypo-vitamin D is an independent risk factor for total mortality in the overall population [52]. Indeed, the adverse effects of vitamin D deficiency are widespread. In particular, playing a pivotal role in several important functions, reduced levels of vitamin D are strictly related to the development and progression of several chronic diseases such as cardiovascular disease, type 2 diabetes, cancer, and depression; moreover, its deficiency may be related to a worsening of bone health and inadequate immune function.

Finally, it is important to note that vitamin D deficiency is often linked to an increased risk of respiratory infections: The latter point could be crucial in viral infections such as COVID-19 [53]. For these reasons, in the scenario of a pandemic infection, although there is still no evidence in the literature that demonstrates with certainty the role of vitamin D in preventing the onset of COVID-19, the use of supplements based on vitamin D has often been discussed as it is believed to play an important role in the prevention of viral infections.

The metabolism and the mechanisms of action of vitamin D are well established [54,55]. Vitamin D3 is produced in the skin by the action of ultraviolet B (UBV) radiation reaching 7-dehydrocholesterol in the epidermis, which is then followed by a thermal reaction. Vitamin D3 or oral vitamin D is converted to 25(OH)D in the liver; subsequently, it is metabolized to the hormone 1,25(OH)2D (calcitriol) in the kidneys. The majority of the effects of vitamin D derive from calcitriol entering the nuclear vitamin D receptor; successively, a DNA-binding protein, interacting with regulatory sequences around target genes, recruits chromatin-active complexes that participate epigenetically and genetically in the modification of transcriptional output [56].

Furthermore, through the detection of the antimicrobial peptide cathelicidin as a vitamin D target gene and the upregulation of VDR in monocytes, vitamin D has been linked to innate immunity. Several studies suggested that cathelicidin activation represents a possible mechanism that drives the protective effects of vitamin D against respiratory tract infections [57]. In addition, in a recent research article, it was shown that vitamin D is also implicated in downregulating the production of pro-inflammatory cytokines, such as interleukin-6 (IL-6), interleukin-8 (IL-8), interleukin-12 (IL12), tumor necrosis factor α (TNF α), and interferon-gamma (IFN-γ), which are involved in the so-called “cytokine storm” [58]. During COVID-19, it has been well reported that the condition of inpatients could be worsening quickly after the cytokine storm: For this reason, the downregulation of IFN-γ and IL-6 inflammatory responses has been reported as negative prognostic markers in critically ill COVID-19 patients [53]. Moreover, considering that vitamin D plays a pivotal role in other important functions such as musculoskeletal activities, its supplementation could be potentially beneficial in the management of COVID-19 patients [59]. Even if further studies should be performed to produce conclusive data on the role of vitamin D in influencing the outcome of COVID-19 patients, collectively this evidence provides a strong rationale for future clinical research.

While contradictory data are available, recent evidence suggests that the use of supplementation with micronutrients may improve the immune response, reducing the risk of infections. In the category of micronutrients, vitamin C [60,61,62,63,64] and the aforementioned vitamin D are included [48,56,65,66,67,68]. In an important recent review [69] the authors state that additional well-designed studies on the role of vitamin supplementations in order to prevent and/or treat COVID-19 infections should be mandatory. Indeed, considering the biological pathways, their dysregulation could be linked to COVID-19 outcomes: For these reasons, further investigation on vitamin C levels at the time of COVID-19 infection, during the infection, and after the infection is required to clarify their role as predictive biomarkers of disease severity. It is important to perform additional investigations focusing on at-risk populations, such as the elderly, pregnant women, immunocompromised subjects, and obese subjects, to define supplementation protocols establishing dosage and duration, for example using vitamin C and/or D. In addition, clinical trials focused on COVID-19 inpatients are needed to provide further evidence on the efficacy of vitamin supplementation as an additional treatment to drug therapy. Conversely, in a recent clinical trial, among hospitalized patients with COVID-19, a single high dose of vitamin D3, compared with the placebo, did not significantly reduce hospital length of stay [70]. The findings do not support the use of a high dose of vitamin D3 for treatment of moderate to severe COVID-19. Moreover, in a cross-sectional/cohort investigation, the authors hypothesized that dietary magnesium alone, particularly its interaction with vitamin D intake, contributes to serum 25-hydroxyvitamin D (25(OH)D) levels, and the associations between serum 25(OH)D and risk of mortality may be modified by magnesium intake level. These authors conclude that it is possible that magnesium intake alone or its interaction with vitamin D intake may contribute to vitamin D status [71]. In this regard, two important clinical trial studies tested the hypothesis that magnesium supplementation differentially affects vitamin D metabolism dependent on baseline 25-hydroxyvitamin D [25(OH)D] concentration. The findings of this studies suggest that optimal magnesium status may be important for optimizing 25(OH)D status [72,73]. Finally, a recent cohort study was performed to determine clinical outcomes of older patients with coronavirus (COVID-19) who received a combination of vitamin D, magnesium, and vitamin B12 (DMB) compared with those who did not. The authors concluded that a vitamin D/magnesium/vitamin B12 combination in older COVID-19 patients was associated with a significant reduction in the proportion of patients with clinical deterioration requiring oxygen support, intensive care support, or both [74].

In this pandemic period, the scientific community has tried to discuss the role that zinc could play in order to prevent COVID-19 infection. As reported in a previous narrative review, the relevance of the oligo element zinc is related to the development and function maintenance of the immune system in all species [75]. In another recent review, as zinc deficiency results in altered numbers and dysfunction of all immune cells, subjects with suboptimal zinc state have an increased risk for infectious diseases, autoimmune disorders, and cancer [76]. To date, based on WHO data, about one-third of the world’s population is affected by zinc deficiency [72]. In this scenario, considering that zinc deficiency accounts for 16% of all deep respiratory infections worldwide [77], in the case of COVID-19 inpatients zinc supplementation it could be indicated in order to reduce the risk of worsening the condition [76,78]. 

Finally, the role of probiotics in preventing viral respiratory infections has also been recently considered. Even though oral supplementation of probiotics is not currently part of any specific protocol for the treatment and prevention of respiratory viral infections, many studies have suggested that their use could be beneficial in the modulation of the systemic immune system. In this way, this modulation may enhance the response to viruses, balancing the inflammatory response [79]. Moreover, analyzing one of the infection pathways of SARS-CoV-2 infection, the gastrointestinal tract is involved, leading to inflammation of the absorptive mucosa and occasionally diarrhea: These two symptoms may exacerbate the immune response, producing mediators of systemic inflammation and worsening the outcome of COVID-19 patients [80,81,82,83,84,85]. For these reasons, the use of probiotic supplementation should be better evaluated.

## 4. Discussion

While there are contradictory data, current research indicates that supplementation with multiple micronutrients could be considered important both in the prevention and in the management of the COVID-19 infection. Particular attention should be paid to the substances that play an important role in the regulation of the immune response, considering the possibility of reducing the risk of infection, and, at the same time, improving the health status of COVID-19 patients. The micronutrients with the strongest evidence for immune support are vitamins C, D, and zinc. To date, evidence has been published about the pivotal role of vitamin D: Its deficiency has been associated with increased susceptibility to respiratory infections. Considering that the main pathway of the SARS-CoV-2 infection is at the lung level, it is reasonable that the use of vitamin D supplements could improve the health status of COVID-19 patients, reducing the risk of infection for healthy individuals, helping COVID-19 survivors in the recovery of their lifestyle. In this way, it is important to consider supplementation in order to improve the recovery in the so-called COVID-19 survivors: With this definition we define a subject who has been infected by the SARS-CoV-2 with a hospitalization period in the ICU, who is still living [81]. The full recovery of COVID-19 survivors, or the implementation of their health status, represents a challenging research field for the scientific community in the near future. 

Moreover, the role of probiotics should be better studied in order to reduce the adverse effects at gastrointestinal levels of the COVID-19 infection. Better design of human clinical trials addressing micronutrient dosing and combinations in different populations is needed to substantiate the benefits of micronutrient overeating against infection.

Moreover, analyzing the data about COVID-19 patients, it is well described that the worst outcomes occur in subjects with one or more comorbidities. Furthermore, each comorbidity is strictly related to metabolic diseases: For example, the overweight or obese subject has a high risk of developing the severe form of SARS-CoV-2 infection. For these reasons, it is important to take into account the influence of lifestyle habits, such as unhealthy diets, on COVID-19 susceptibility and recovery. In addition, the large number of subjects who recover from COVID-19 could lead to a spike in chronic medical diseases. These conditions could be further exacerbated by a poor diet regimen. Therefore, in consideration of the data discussed in this review, it should be recommended that subjects should avoid eating foods containing high amounts of saturated fat and sugar; contrariwise, it is desirable that they consume high amounts of fiber, whole grains, unsaturated fats, and antioxidants to enhance immune function [1,86].

Finally, during the pandemic period, the introduction of different countermeasures such as the “lockdown”, long-term quarantine in cases of suspected or confirmed COVID-19, could generate the adoption of unhealthy eating habits, increasing the risk of non-communicable diseases in the middle-long term.

Even if several measures such as the assumption of a specific diet regimen, the use of dietary supplements, and other similar interventions are promising for the prevention, management, and recovery of COVID-19 patients, it is important to highlight that strong data from randomized clinical trials are needed to support any such assumption.

## Figures and Tables

**Table 1:** The selected articles analyzed in this review with the relative main findings are summarized.

Authors	Geographical Area	Type of Article	Main Findings
Calder et al. 2020 [3]	Europe (UK, Netherlands), USA, New Zealand	Review	Encouraging public health to include nutritional approaches, improving public health, and reducing the impact of known and unknown viral infections.
Stachowska et al. 2020 [24]	Poland	Systematic review	Subjects with a mild course of the infection should be additionally supported for nutritional status, particularly the elderly or polymorbidity subjects.
Cena et al. 2020 [25]	Italy	Mini review	The choice of dietary regimes that can potentially function as a supplement to prevent undesirable effects, such as hyperinflammation, could be beneficial for patients with mild signs of SARS-CoV-2.
Laviano et al. 2020 [26]	Italy	Editorial	Different studies show that COVID-19 is linked to adverse outcomes in elderly and comorbid patients with a hypoalbuminemia status. These features are not particular to the Chinese population considering that this status has been described in North American patients with COVID-19 as well. The recent literature on COVID-19 patients indirectly underlines the importance of diet in determining the health status of COVID-19 patients.
Arkin et al. 2020 [27]	USA	Letter to editor	The authors suggested providing recommendations and guidelines to increase patient safety, providing awareness about this important theme. Particularly, based on the data collected worldwide, there should be an effective way to provide diet recommendations.
Correia 2020 [28]	Brazil	Review	This review highlighted the role of nutrition as an integral part of each person’s health care. For this reason, it should be important to act both on medical care and on diet regimen for COVID-19 patients. Moreover, it is crucial to understand the comorbidities of COVID-19 patients (they were frequently affected by several diseases linked to metabolic problems) that are associated with adverse effects and, in about 2% of cases, with death.
Aman and Masood 2020 [29]	Pakistan	Brief communication	The authors focused on the role of vitamin C as an immune booster. Moreover, they highlighted that an optimal diet status is almost certainly linked to a healthy immune system. For these reasons, they strongly encourage the adoption of dietary management guidelines to support the health status of COVID-19 patients.
Mehta 2020 [30]	UK	Rapid report	Considering that the COVID-19 fight is critical in different aspects, this study suggested that the nutritional needs of the population are met and sustained, including those who are most vulnerable. Prevention, diagnosis, and treatment of malnutrition must also be included in the routine management of COVID-19 inpatients.
Naja and Hamadeh 2020 [31]	Lebanon	Perspective	There is still much to be known about the SARS-CoV-2 infection; particularly, scarce knowledge about the relationship between nutritional status and COVID-19 infection has been published. In this scenario, it is important to clarify several aspects of the role of the diet regimen.
Patel et al. 2020 [32]	USA	Review	In this review, the authors focused on the major aspect of the management of the COVID-19 inpatient. Particularly, they highlighted the role of nutrition during ICU stay.
Kelea and Klimis–Zacas 2020 [33]	USA	Editorial	In this editorial, the authors explore new evidence related to the non-pharmacological treatment to reduce the adverse effects of COVID-19 and to prevent infection.
Lidoriki et al. 2020 [34]	Greece	Brief communication	In this brief communication, the authors report that the nutritional status of COVID-19 patients is strictly related to the patients’ outcome. For example, elderly people, who are usually characterized by malnutrition status, are more vulnerable to the infection and to its complication; similarly, obese subjects have the worst outcome compared to normal weight subjects, after SARS-CoV-2 infection.
Liu et al. 2020 [35]	China	Article	The main findings of this article showed that strengthening the nutritional status of COVID-19 inpatients may improve disease outcomes. In this way, the authors suggested increasing this aspect in COVID-19 management.
Budhwar et al. 2020 [36]	India	Review	In this review, the authors suggested that the consumption of immunity-boosting foods may help prevent respiratory disorders or suppress disease-related problems, which could be useful in monitoring the diffusion of the SARS-CoV-2 infection. Finally, they suggested improving a specific dietary intervention for each infected subject, particularly, before starting generalized treatments and interventions.
Fernández–Quintela et al. 2020 [5]	Spain	Review	In this review, the authors pointed out the role of the nutritional aspect in the COVID-19 infection, encouraging future studies to clarify the value of specific diet regimens realizing descriptive studies and/or randomized controlled trials.
Hakeem and Sheikh 2020 [37]	Pakistan	Review	In this review, the authors focused on the nutritional role as an important factor in order to reduce mortality and morbidity in different COVID-19 patients.

## Data Availability

Data sharing is not applicable to this article.
